# Treatment of Midshaft Humerus Fractures Using Early Functional Bracing: Results and Prognostic Factors

**DOI:** 10.7759/cureus.14852

**Published:** 2021-05-05

**Authors:** Georgios Arealis, Giles Faria, Milan Kucera, Cosmin Crisan, Sathya Murthy

**Affiliations:** 1 Orthopedic Surgery, East Kent Hospitals University NHS Foundation Trust, Canterbury, GBR

**Keywords:** midshaft humerus fractures, functional bracing, prognostic factors, radiographic measurements

## Abstract

Purpose

Our study tests the hypothesis that a new radiographic measurement, the fracture displacement index (FDI), is associated with the prediction of successful conservative treatment, and investigates factors, that contribute to failure treatment.

Methods

This was a retrospective case series reporting the results of pragmatic treatment of midshaft humerus fractures using a humeral brace. Details regarding the patient demographics and fracture pattern were recorded. The outcome was measured as patient satisfaction, return to activities, and need for further treatment at a minimum of one year from the discharge date.

Results

Of the 33 surviving patients, who met the inclusion criteria, two subgroups were developed. The conservatively treated brace group (n=23, 70%) and the surgical group (n=10, 30%). The median age of brace group patients was 48.3 years, significantly less (p=0.0025) than the surgical (72 years). There were no significant differences in the rest of both group demographics. On the first available x-ray after the brace application, there was a significant difference in FDI (p=0.001) between groups. Residual angulation was significantly better for the surgical group. Skin breakdown was the most common complication, followed by forearm swelling. Stiffness was common in both groups.

Conclusion

Patients with FDI near 50 younger than 60 years have better chances to proceed to union if treated with brace less than 24 hours after the injury. Patients with FDI larger than 100, older than 78, have a higher risk of requiring surgery. All patients should be counseled about the risk for skin complications and developing forearm swelling.

## Introduction

Since it was first reported by Sarmiento et al. [1] functional bracing has become the gold standard for treating humeral shaft fractures conservatively. Most studies report good to excellent results in more than 80% of patients with a high union rate (94.5%) [2].

Statistical analysis has shown that proximal third fractures and AO type A fractures have a higher non-union rate although this has now been proven to be not statistically significant. Until now, no radiographic measurement has been proven to be useful for the prediction of progression to union, except for fragment gap in AO type A fractures [3]. Patient factors such as sex, obesity, and smoking have been shown to be predictors of union [2].

Our study presents a new protocol of treatment using only humerus brace and tests the hypothesis that a new radiographic measurement, the fracture displacement index, is associated with the prediction of successful conservative treatment. Additionally, we investigated if other factors, patient or fracture-related, could be contributing to failure of conservative treatment with a brace.

## Materials and methods

This is a retrospective case series reporting the results of pragmatic treatment of midshaft fractures of the humeri using humeral braces. Following approval by the research ethics committee of our organisation, and using the trauma database, we identified all the patients that sustained a midshaft humeral fracture from January 2017 to March 2019. Inclusion criteria were patients of all ages that were treated with a humeral brace. Open fractures were excluded. Patients were also excluded if they had sustained a pathological fracture, had vascular injury and were not suitable for brace treatment. These included serious skin condition, lymphedema and ipsilateral breast cancer treated with lymph node excision.

The detailed records and X-rays of all patients meeting the eligibility criteria were retrieved and reviewed. Details regarding the patients’ date of birth, mechanism of injury, radial nerve palsy on presentation, type and exact position of fracture, medical history, intervention, X-ray measurements, as well as any complications of treatment were recorded. Additionally, outcome was measured as patient satisfaction, return to usual activities and need for further treatment at a minimum of one year from the discharge date.

Brace application

**Figure 1:**
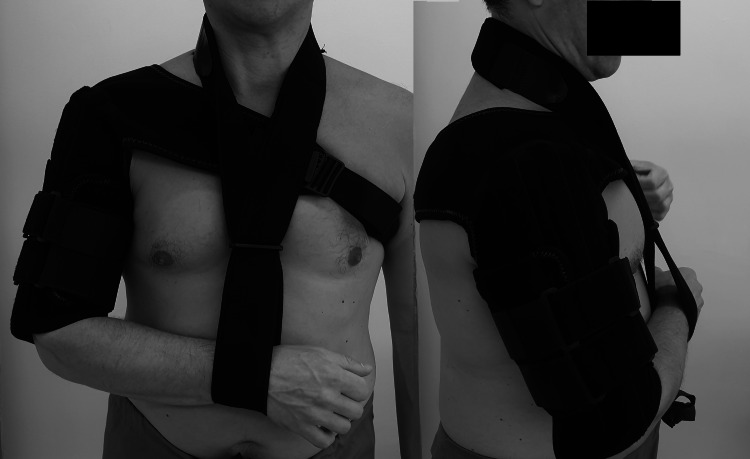
The humeral brace used for conservative treatment (Clasby Humeral Brace, Beagle Orthopaedics, Blackburn, UK).

Once a midshaft closed humerus fracture was identified, and after the radial nerve function was assessed and documented, a prefabricated brace was applied (Figure 1, Clasby Humeral Brace, Beagle Orthopaedics, Blackburn, UK). All patients were treated with the same commercial brace. This was to be applied within 24 hours, either in the plaster room or out of hours, by a trained nurse practitioner in the emergency department (ED) or at a minor injury unit (MIU). If no brace was available in ED and MIU then a broad arm sling, with a body strap was used, and the brace was applied in the plaster room the following day. Post brace application, the radial nerve was reassessed and if palsy developed then the brace was removed. Post-brace check X-ray was performed on every patient. Every case was reviewed, and the final plan was made by a senior orthopaedic surgeon within 72 hours. If the patient was for continued brace treatment, then an appointment in one week was made in order to check brace fitting, assess the skin and perform a check X-ray to confirm the acceptable position of the fracture in the brace. The patient was followed up until fracture healing or non-union developed and surgery was deemed necessary.

Surgical procedure

Once the patient and radiographs were reviewed by a senior orthopaedic surgeon, a decision was made to continue with brace or plan for surgical treatment. Overall, there were two groups of surgical patients; those that required early surgery (due to significant displacement or patient preference) and those that had delayed surgery (due to fracture non-union). The surgical procedures were performed by a typical method. Of the early surgery patient group, five were treated with an intramedullary nail and the remaining one patient, along with the delayed surgery group, underwent an open reduction and internal fixation with a plate.

Radiographic measurements

**Figure 2:**
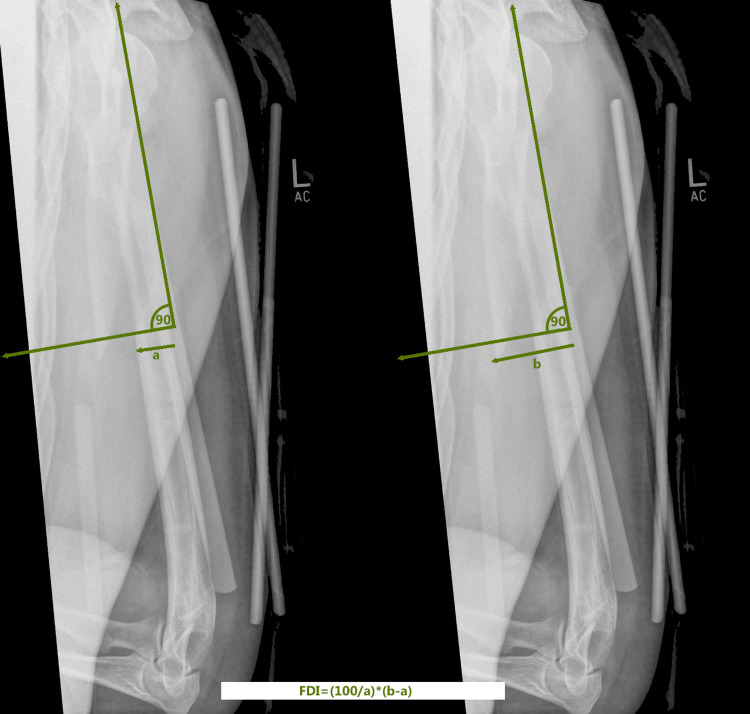
Method of measurement of the fracture displacement index (FDI).

X-rays were reviewed using the hospital PACS system and measurements were made. On the initial post-injury X-ray, the following features were determined: the fracture pattern according to AO, presence of butterfly fragment, position of fracture on the diaphysis, presence of a second fracture, distraction and initial angulation in two planes. On the first X-ray after the brace application, the fracture displacement index (FDI) was calculated. The FDI was defined as the percentage of displacement compared to the diameter of the bone at the level of the fracture. This was calculated using a line vertical to the shaft of the humerus, at the level of the greatest displacement and using the equation: 100 divided by bone diameter and multiplied by fracture displacement [total width (displacement and bone diameter) minus bone diameter] (Figure 2). This allowed for the displacement to be calculated as a percentage and avoid the errors arising from X-ray magnification. For this reason, we did not require calibrated radiographs and specific imaging protocol. We calculated the FDI in both planes and used the highest value. On the final X-rays final distraction, shortening and angulation in two planes were measured.

Statistical analysis

Descriptive statistics were performed for all variables measured, including kurtosis and skewness. Quantitative variables were calculated as median and standard deviation, or median and range, while qualitative variables were presented as frequency counts and percentages. The difference between medians of quantitative data was measured using non-parametric tests (Mann-Whitney U). Quantitative data were assessed using Chi-squared tests (Pearson and Fisher’s). A p-value of <0.05 was considered statistically significant. Statistical analyses were performed with the SPSS software (version 26, IBM, Armonk, NY).

## Results

Forty patients were assessed for eligibility to enter the study. A total of five patients were excluded, two because they were out of the area and could not be followed up, one had pathologic fracture, one had proximal and one distal humeral fracture. This left 35 patients who met the inclusion/exclusion criteria of the study. Two patients (5%) were deceased at the time of the study, however available data from their records were included in demographics but not in the final statistical analysis. Both patients died from unrelated causes due to serious co-morbidities. One patient (medical history: anemia of unknown origin, atrial fibrillation, and hypothyroidism) died two months after the injury and 44 days after surgery. This patient could not tolerate the brace and was therefore treated with an intramedullary nail. The other patient died three months after injury (medical history: alcoholism). This patient was listed for surgery, as there were no signs of union but died soon after.

The median age of all the patients was 57 years (SD:+/- 21.8), 51% were female and the right side was involved almost half of the time (45.7%). Median time from injury to discharge was 109 days (range: 29-476). All patient demographics are in Table 1.

**Table 1:** Patient characteristics.

	All patients (n=35)	Brace group (n=23)	Surgical group (n=10)	
Age, y, median (SD)	57±21.8	48.3±22.1	72±15	p=0.025
Sex, female, n (%)	18 (51%)	12 (57.2%)	5 (50%)	p=0.909
Side of injury, right, n (%)	16 (45.7%)	9 (39.1%)	6 (60%)	
Median time from fracture to discharge, days (range)	109 (29-476)	85 (42-476)	197 (29-434)	p=0.025
Diabetic, n (%)	3 (8.6%)	1 (4.3%)	2 (20%)	p=0.151
Osteoporosis (28 patients with records), n (%)	9 (25%)	5 (21.7%)	9 (90%)	p=0.001
Significant co-morbidities, n (%)	24 (67%)	14 (60.9%)	8 (80%)	p=0.284
Fall on arm as mechanism, n (%)	21 (60%)	12 (52%)	8 (80%)	p=0.133
Radial nerve injury on presentation, n (%)	2 (5.7%)	2 (8.7%)	0 (0%)	

Of the 33 surviving patients, two subgroups were developed. The conservatively treated brace group (n=23, 70%) and the surgical group (n=10, 30%). From the surgical group, four patients (12%) had delayed surgery due to non-union and six (18%) early surgery. The reasons for early surgery were either patient not willing or able to tolerate the brace or significant fracture displacement. The median age of brace group patients was 48.3 years (SD:+/- 22.1) and this was significantly less (p=0.0025) than the surgical group (72 years, SD+/-15). Median time from injury to discharge for the brace group was 85 days (range: 42-476) and this significantly less (p=0.025) than the surgical group (197 days, range: 29-434). There were no significant differences in the rest of both group demographics that are presented in Table 1.

The main mechanism of injury was fall on the arm. This was more common in the surgical group (eight patients, 80%) than in the brace group (12 patients, 52%), but this was not statistically significant (p=0.133). Osteoporosis was significantly (p=0.001) more common in the older surgical group (nine patients, 90%) than in the brace group (five patients, 21.7%). All patients had significant comorbidities (brace group 60.9% and surgery group 80%, p=0.284) these ranged from psychiatric to serious heart conditions and stroke (Table 2). 

**Table 2:** Patient co-morbidities.

Group	Comorbidity	Frequency	Percent
Brace	Alcoholic, epileptic, ex-iv drug user	1	4.3
	Anaemia, cirrhosis	1	4.3
	Autism, asthma	1	4.3
	Crohn’s, depression, alcoholic, multiple overdoses	1	4.3
	Chronic, lymphoid leukemia, Parkinsonism, stroke	1	4.3
	Chronic obstructive pulmonary disease (COPD), pulmonary embolism (PE)	1	4.3
	Depression, anxiety	1	4.3
	Depression, anxiety, suicidal	1	4.3
	Deep vein thrombosis (DVT)	1	4.3
	Epileptic, Korsakoff’s psychosis	1	4.3
	Multiple sclerosis, mobilizing with rollator	1	4.3
	Parkinson's, Lewy body disease	1	4.3
	Prostate cancer, COPD, hypertension, hypothyroid	1	4.3
	Type 1 diabetic, hypertension	1	4.3
Surgery	Crohn’s, hypertension	1	10
	Diabetes type 1, hypertension, ischaemic heart disease, osteoporosis under treatment	1	10
	Diabetes type 2, hypertension	1	10
	Hypertension	1	10
	Neck of femur fracture (cannulated screws), hypothyroid, AF, dementia	1	10
	Right breast cancer 2010, anastrozole	1	10
	Sarcoidosis, osteoarthritis shoulder	1	10
	Spinal fusion, chronic backpain, fibromyalgia	1	10

Radiographic analysis

**Figure 3:**
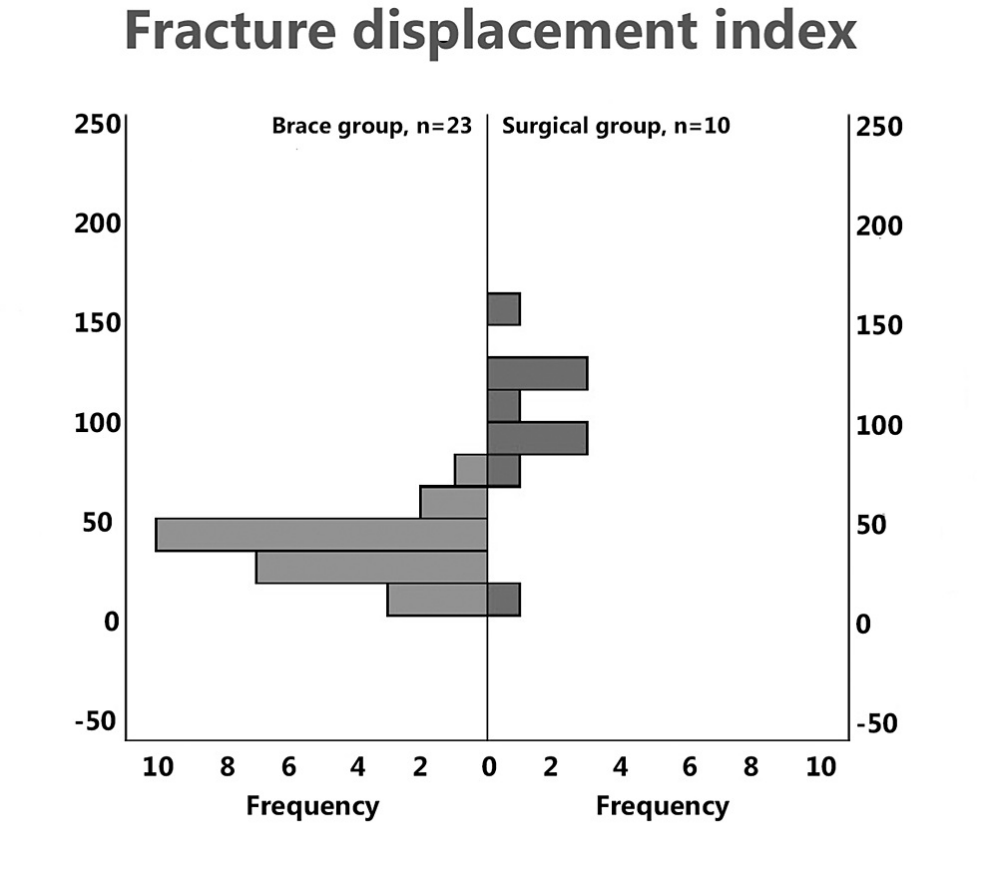
The number of patients treated in brace (left) and with surgery (right) and their fracture displacement index.

On the initial injury X-rays, fracture pattern and type were assessed. The most common pattern was spiral for both groups (brace: 14/23 patients-60.9%; surgery: 8/10 patients 80%, p=0.669) and AO type A1 (brace: 6/23 patients-26.1%; surgery: 8/10 patients 80%, p=0.57). A large butterfly fragment was present in eight patients from the brace group (34.8%) and one from the surgical group (10%, p=0.217). The most common fracture area was the middle of the shaft (brace: 13/23 patients - 56.5%; surgical 4/10- 40%, p=0.318) and finally, three patients from the brace group (13%) and two from the surgical (20%, p=0.627) had a second fracture either proximal or distal. Overall, there were no statistically significant differences in fracture configuration and classification between the two groups (Figure 3, Table 3).

**Table 3:** Fracture configuration and classification.

Group	Configuration (p=0.669)			AO type (p=0.57)			Butterfly (p=0.217)			Thirds (p=0.318)
		Frequency	Percent		Frequency	Percent		Frequency	Percent	
Brace	Spiral	14	60.9	A1	6	26.1	No	15	65.2	Proximal
	Oblique	3	13	A2	3	13	Yes	8	34.8	Middle
	Transverse	4	17.4	A3	3	13				Distal
	Comminuted + segmental	2	8.7	B1	2	8.7				Combined proximal middle
				B2	1	4.3				
				C1	6	26.1				
				C2	2	8.7				
Surgery	Spiral	8	80	A1	4	40	No	9	90	Proximal
	Oblique	1	10	A2	1	10	Yes	1	10	Middle
	Transverse	1	10	A3	1	10				Combined proximal middle
				B1	2	20				
				C1	1	10				
				C3	1	10				

On the first available X-ray after the brace application, we measured the fracture displacement index (FDI), there was a significant difference (p=0.001) between the brace (median 41, range 4-47) and the surgical group (104, range 0-153).

Of all the other measurements almost all were similar except for residual angulation that was significantly better (p=0.001) for the surgical group (median 2 degrees, range 0-7) than the brace (median 9 degrees, range 4-75). Residual shortening was borderline better (p=0.051) for the surgical group (median 0 mm, range 0) than the brace (median 0 mm, range 0-15). All measurements are presented in Table 4.

**Table 4:** Radiographic measurements.

	Brace (n=23)	Surgery (n=10)	p
Distraction (median mm, range)	0 (0-12)	0 (0-7)	0.384
Initial angulation AP (median degrees, range)	5 (0-25)	6.5 (1-25)	0.773
Initial angulation lateral (median degrees, range)	10 (0-25)	9.5 (2-25)	0.985
Fracture displacement index (median, range)	41 (4-75)	102 (0-153)	0.001
Residual angulation AP (median degrees, range)	9 (0-40)	2 (0-7)	0.001
Residual angulation lateral (median degrees, range)	4 (0-25)	3 (0-7)	0.55
Residual shortening (median mm, range)	0 (0-15)	0 (0)	0.051
Residual distraction (median mm, range)	0 (0-8)	0 (0)	0.714

Radial nerve injury (Table 5)

Radial nerve palsy on presentation was similar between groups (p=0.479). In the brace group, two patients (5.7%) presented with radial nerve palsy, both were treated with brace and made full recovery. None of the patients in the surgical group presented with radial nerve palsy.

Radial nerve injury as complication of treatment was similar between groups (10%, p=0.303). No patient developed radial nerve injury after the application of the brace. One patient, treated with nail, developed postoperative radial nerve palsy. This required nerve release, as the nerve was identified to be compressed between the fracture fragments and the patient made a full recovery.

Complications (Table 5)

Both brace treatment and surgery resulted in different significant complications. Infection occurred in one surgical patient (10%, p=0.303). Breakdown of the skin occurred in one surgical patient (10%) and three patients in the brace group (13%, p=0.689). Forearm swelling was observed in three surgical patients (30%) and 6 brace patients (26.1%).

The most important complication of brace treatment was shoulder and elbow stiffness requiring prolonged physiotherapy that occurred in almost half of the patients (12 patients, 52.2%). This was less, but not significantly, for the surgical group (three patients, 30%, p=0.283).

**Table 5:** Complications.

Complication (n, %)	Brace (n=23)	Surgery (n=10)	p
Radial nerve on presentation	2 (8.7%)	0 (0)	0.479
Radial nerve complication	0 (0)	1 (10%)	0.303
Radial nerve residual	0 (0)	0 (0)	
Stiffness and extended physio	12 (52.2%)	3 (30%)	0.283
Infection	0 (0)	1 (10%)	0.303
Skin complications	3 (13%)	1 (10%)	0.689
Forearm swelling	6 (26.1%)	3 (30%)	0.685

Follow up and final results 

The median time from injury to brace application was significantly less (p=0.038) for the brace group (0 days, range 0-18) compared to the surgery group (five days, range 0-23). All the patients in the brace group proceeded to union. The surgical group had four patients (4/33 patients, 12.1%) that required surgery for non-union following brace treatment at a median of 97 days (range 44-161). Of the remaining six patients of the surgical group, two (2/33 patients, 6%) had surgery due to patient preference (not consenting for brace treatment), two had other fractures that required surgery (one neck of femur and one distal radius), and a decision was made to operate on all injuries and the remaining two had significantly displaced fractures.

The patients in the brace group required to remain a median of 53 days in brace (range 33-104). This was more, but not significant (p=0.060), than the 28 days of the surgery group (0 to 94). The surgery group took longer to discharge (median 197 days, range 29-434, p=0.025) than the brace group (85 days, range 42-476).

We must consider that the treatment period is quite lengthy and some of the patients were followed up for more than a year. Additionally, a large proportion of our patients had significant comorbidities (brace group 60.9% and surgery group 80%, p=0.284). These two factors make recovery slower and focus the treatment on achieving daily activities as the main goal. All our patients returned to their daily activities and were pleased with the result of the treatment. At a minimum of one-year post-discharge, none required a new appointment and any further treatment for their injuries (Table 6).

**Table 6:** Follow up and final results.

Group		Days to brace (p=0.038)	Days to non-union	Days in brace (p=0.060)	Days to discharge (p=0.025)
Brace	n	23	23	23	23
	Median	0	0	53	85
	Minimum	0	0	33	42
	Maximum	18	0	104	476
Surgery	n	8	4	8	10
	Median	5	97	28	197
	Minimum	0	44	0	29
	Maximum	23	161	94	434

Prognostic factors for successful treatment in brace 

Analysing all the data collected and using one way ANOVA we identified three significant prognostic factors age (F(1,31)=5.82, p=0.022), fracture displacement index (F(1,31)=36.7, p=0.001) and time from fracture to brace (F(1,31)=6.3, p=0.017). When we entered all the above in a regression of categorical data model the overall fit was adjusted R^2^ = 0.892. The main predictor was the displacement index (β=0.914, p=0.001) with days from injury to brace the second (β=0.251, p=0.48) but age was not found to contribute significantly (β=-0.104, p=0.269). Therefore the most important prognostic factor was the fracture displacement index; using Kaplan-Meier analysis we identified that FDI of median 48 (95%CI 41 to 51) was the best predictor for union and median 104 (95%CI 90 to 117) was predictive of need for surgery. Similarly, median age leading to union was 61 (95%CI 49 to 72), and to need for surgery was 78 (95%CI 70 to 85). Lastly, median days to brace application for union was 1 (95%CI 0.3 to 1.6)

Combining all the above we concluded that patients with FDI near 50 and younger than 60 years old had better chances to proceed to union if treated with brace less than 24 hours after the injury. Patients with FDI larger than 100, especially if older than 78, have a higher risk of requiring surgery.

## Discussion

The treatment of midshaft humeral fractures with brace was initially proposed by Sarmiento in 1977 [1]. Since then it has become the first choice of treatment for many authors [2] as it has been proven to be successful, non-invasive, and cost-effective [2,4,5].

Most papers suggest application of brace after a week [1,6] or two weeks [7] at least when the swelling has settled down [6] after an initial period of treating the fracture in a cast of slab [1,6-8]. In our series, the brace was applied as soon as possible after the injury with a median for the brace group of 1 day and for the surgical group of 5 days. The time to brace application was found to be very important for the progression to union (ANOVA (F(1,31)=6.3, p=0.017, regression (β=0.251, p=0.48). We were able to avoid the need for an interim period in cast as we used a brace made of fabric with the ability to adjust to the arm swelling. One more advantage of the direct brace application was that it is easier to fit and reduces the need for a change of treatment after a week or two.

Non-union rates described in the literature vary from 2.8% [1] to 39.6%[8] with an overall rate of 5.5% [2]. Few theories for the discrepancies have been described. These include poor follow up rates, leading to amplification of proportions of non-union patients, who will present back to follow up more reliably than healed unions [9-11]. In addition, differences in protocols of applying a humeral brace across different countries and institutions have been observed in many studies, such as the use of U-slab, hanging cast, or backslab initially (and for different periods of time) before brace application, all of which could affect final outcomes [8,12,13]. In the literature, the reported time to union varies 8 to 30 weeks with an average of 10.7 weeks [1,2,12,14,15]. In our series the non-union rate was 12.1% for the patients treated initially with brace and the median time to non-union was 97 days (13 weeks). Our results are similar to the ones reported in literature, with our non-union rate being slightly higher, but this can be explained from our brace protocol that included use of brace for all patients presenting to the emergency department.

A recurring theme suggests that when humeral brace is used to treat open fractures, there appears to be a slightly longer average time to healing [10,16] with mean of 13.6-14 weeks for open fractures [2]. In our series, we did not encounter any open fractures, but they would be excluded from the study as per protocol.

Various patient factors include female sex, smoking, obesity, non-steroidal anti-inflammatory drug use [3-5,8,17,18] have been reported to contribute significantly to non-union. In our series: sex, diabetes, and serious comorbidities were not identified as factors contributing to non-union. We were not able to assess smoking and steroid use as this was a retrospective study, and our patient’s comorbidities meant that many were not smoking, but both have been proven to contribute significantly to fracture union. Osteoporosis was less common in the brace treatment group (p=0.001) but we cannot confirm that this is a reason for successful union as the median age of this group was 57 years and this was significantly less than the surgical group (72 years, p=0.025). In the ANOVA age (F(1,31)=5.82, p=0.022) was found to be a significant predictor of union but it didn’t contribute as much to the regression model ((β=-0.104, p=0.269).

Proximal oblique fractures and mid-diaphyseal transverse and short oblique fractures have a reported a higher risk of non-union, similar to AO type A fractures that can lead to a 20% non-union rate [3,17]. This risk has not been proven to be statistically significant for any fracture configuration [2,19] and this is consistent with our findings. We could not identify a fracture pattern that can reliably predict fracture non-union.

The gap between fragments at the time of injury has also been postulated as a potential factor for non-union, with Neuhaus et al. reporting that every millimeter gap increased the risk of fracture mobility six weeks after injury by about 40%, either because of the larger bridge required for the callus, but this also may represent greater soft-tissue injury. [3] Replicating the gap measurements, was difficult due to X-ray scale and calibration and due to errors, as the measurement in mm is prone to user error. We developed the FDI as a measurement that is independent of scale and calibration, as it is a percentage, and it is measured in a larger scale allowing for user error. This was found to be the most significant predictor of fracture healing both using ANOVA (F(1,31)=36.7, p=0.001) and in the regression model (β=0.914, p=0.001). An FDI below 50 was a good predictor of union and above 100 of non-union (Figure 3). Two more factors, time from fracture to brace (F(1,31)=6.3, p=0.017) and age (F(1,31)=5.82, p=0.022) were also identified, with age playing a lesser role in the Kaplan-Meier (KM) regression analysis.

Radial nerve palsy on presentation should always be assessed and recorded but it does not consist an absolute contradiction to bracing. In the literature, the reported incidence of radial nerve palsy after humeral fracture can be 2%-22% [2,14]. In the majority of cases, the nerve is intact and 95% will go on to full recovery within three months [2,14] however some surgeons feel that radial nerve palsy after injury was an indication for immediate exploration [2,14]. In our series, two patients (5.7%), presented with palsy but both made full recovery at two months post-injury. None of our patients developed palsy after the application of the brace, this would have been an indication to disuse the brace and proceed to surgery for our institution. One patient, treated with nail, developed postoperative radial nerve palsy (10%, p=0.303) that fared well after surgical decompression.

Malunion, especially varus angulation, is a common complication, more often observed after conservative treatment. The original Sarmiento paper quotes an average of 4 degrees varus angulation. In his study, 15.67% of patients exhibited 10-20 degrees [1]. Since then, usual angulation reported is less than 10 degrees but on some occasions, up to 20 degrees are noted [2,10-12,15,16]. The average is 5.6 degrees [2]. Obesity (although conversely, obesity may be a risk factor for varus angulation adipose tissue hides the deformity well) does not generally affect function with most obese patients not showing any clinical deformity and recovering full physical function, although displaying a greater radiograph angulation pattern [5]. In our series, median residual angulation in the brace group was 9 degrees similar to the reported in the literature, and as expected this was significantly worse than the surgical group (median 2 degrees, p=0.001). One of the patients in the brace group had a residual angulation of 40 degrees, the patient missed almost all of the follow-up appointments but continued using the brace, at final follow up he was functional and returned to activities despite the significant angulation. Residual angulation in the lateral, sagittal, plane is less significant in all reported series, with a reposted average of 3.7 degrees [2]. Our results are again similar, with a median of 4 degrees in the lateral views. This was similar to the surgical group (median 3 degrees, p=0.55). We did not measure rotational deformity, as it has not been proven to be a significant issue and requires CT scan for accurate measurement [2,20]. Shortening of the humerus after fracture healing has not been assessed in most published papers but is usually minimal, less than 20 mm and no clinical significance has been reported as a result of this [2,15,16]. Similarly in our series, the median shortening was 0 mm but the range was up to 15 mm in the brace group.

Shoulder and elbow stiffness is a common complication following treatment with a humeral brace [2,4,10,11,15,21]. This can require prolonged physiotherapy but usually recovers with return to use. In our series, 52% of the patients treated with a brace required extended physiotherapy in order to return to function and this was more than the surgical group, but not significantly (30%, p=283).

Accounting for the cost of surgery, inpatient treatment and follow-ups, including treatment of infection and nerve complications that are more common after surgery it has been reported that treatment with a brace is more cost-effective, accounting for almost half of the costs of surgery [22,23]. We did not measure the cost of either treatment in our series, but we postulate that using a brace-only treatment protocol reduces the overall cost as fewer consumables are used and there is no need to change the method of treatment from cast or backslab to brace.

Overall complication rates are very low following brace treatment, with most papers reporting skin irritation as the most common. This resolved with skincare and adjustment of brace [15,16]. In our series, three patients (13%) in the brace group and one (10%, p=0.689) in the surgical group developed skin complications. We were strict with the brace protocol and an appointment was made within a week to assess the skin. One very common complication in all groups was forearm swelling that was experienced by 26% of the brace patients and 30% of the surgical patients. This resolves over the period of the treatment but patients should be informed that this is expected in order to avoid frustration (Table 7).

**Table 7:** Guidelines for diaphyseal humerus fracture brace. ED: emergency department; MIU: minor injury unit.

ED/MIU/admitting team	Follow-up
Confirm closed fracture	Skin complications should be minimal
Test and document radial nerve function	First appointment in 1 week (skin check and check XR)
Brace applied within 24 hours (ED or MIU or Plaster Room- If no brace available in ED use broad arm sling with body strap )	Case reviewed and plan made by senior orthopaedic surgeon within 72 hours
Post-brace radial nerve re-assessment: remove brace if changed	
Post-brace check XR on every patient	
Refer to orthopaedic SHO on call	
Provide relevant leaflet and contact details	

The evaluation of the results of treating humerus midshaft fractures with a brace, is not uniform. Most studies report excellent and good results in more than 80% of the patients [2]. Our results are very similar to the ones reported in the literature. Despite the fact that the follow-up period was lengthy, and a large number of patients required extensive physiotherapy, all our patients returned to their activities and were pleased with the results. This happened even though a large proportion of them had serious comorbidities. Additionally, at a minimum of one year after discharge none of our patients required a new appointment and further treatment.

Strengths of our study include the extensive follow up period, which was more than one year, at minimum, from discharge from clinic. Second, is the development of a measurement that is independent of x-ray calibration and is coarse to allow for user error in measurements. Nevertheless, it should be interpreted with its limitations. First, it is a retrospective study, based on patient records and medical database inputs. Second, the sample number of our patients is small, especially the ones that required surgery. Third, four patients (11%) did not have an X-ray immediately post brace application and we used the first available X-ray from the one-week appointment for X-ray measurements. 

## Conclusions

This study tested the hypothesis that a new radiographic measurement, the FDI can be used as a predictor of successful conservative treatment of midshaft humerus fractures with a brace. The advantage of the FDI measurement being that no calibration or specific imaging protocol is required, and it can be calculated using standard X-rays. Additionally, it investigates other factors, patient or fracture-related, that can contribute to failure of conservative treatment. In summary, our findings suggest that patients with FDI near 50 and younger than 60 years old have better chances to proceed to union if treated with a brace less than 24 hours after the injury. Patients with FDI larger than 100, especially if older than 78, have a higher risk of requiring surgery. All patients should be counseled about the risk for skin complications and developing forearm swelling.
